# T-cells contribute to hypertension but not to renal injury in mice with subtotal nephrectomy

**DOI:** 10.1186/s12882-017-0555-0

**Published:** 2017-05-08

**Authors:** Nynke R. Oosterhuis, Diana A. Papazova, Hendrik Gremmels, Jaap A. Joles, Marianne C. Verhaar

**Affiliations:** Nephrology & Hypertension, University Medical Center Utrecht, PO Box 85500, 3508GA Utrecht, Netherlands

**Keywords:** Albuminuria, High salt diet, Hypertension, Subtotal nephrectomy, T-cells

## Abstract

**Background:**

The pathological condition of chronic kidney disease may not be adequately recapitulated in immunocompromised mice due to the lack of T-cells, which are important for the development of hypertension and renal injury. We studied the role of the immune system in relation to salt-sensitive hypertension and renal injury in mice with subtotal nephrectomy (SNX).

**Methods:**

Wild-type immunocompetent (WT) and Foxn1^nu/nu^ athymic immunodeficient (AT) CD-1 mice underwent SNX to induce renal injury after which they received standard chow or a high salt diet (HSD). Four weeks after SNX blood pressure and kidney function parameters were measured.

**Results:**

HSD increased albumin excretion independent of immune status. Systolic blood pressure increased only in WT mice on HSD, not in AT mice. Uremia and morphological damage after SNX were not affected by either HSD or immune status.

**Conclusions:**

For the development of hypertension after SNX in CD-1 mice mature T-cells and a high salt diet are required. SNX induced albuminuria was independent of the presence of T-cells.

**Electronic supplementary material:**

The online version of this article (doi:10.1186/s12882-017-0555-0) contains supplementary material, which is available to authorized users.

## Background

Cell-based therapy is a promising treatment approach for many degenerative pathological conditions and has been proposed as potential therapeutic strategy in chronic kidney disease [1]. Immunocompromised mice, lacking an adaptive immune system, are often used in pre-clinical studies to test the therapeutic effects of human stem cells [2]. However, the adaptive immune system is also involved in the development and maintenance of hypertension and renal injury [3]. Immunocompromised animals may therefore not adequately resemble the pathological condition of chronic kidney disease (CKD).

In rats with CKD induced by subtotal nephrectomy (SNX), elimination of T-cells by thymectomy or splenectomy prevented the development of hypertension, while transfer of lymph node cells into SNX rats induced hypertension [4]. Angiotensin-II (AngII) infusion in SCID (severe combined immunodeficiency) mice resulted in lower SBP, albumin excretion and renal damage than in wild-type mice [5] and hypertension was not maintained after subtotal nephrectomy in athymic mice [6]. Moreover, immunosuppressive drugs reduce salt-sensitive hypertension and renal inflammation caused by protein overload [7] or ischemia/reperfusion injury [8].

The 5/6th nephrectomy ablation model is a well-known experimental model of progressive renal disease that resembles many aspects of human CKD, including hypertension, and would be valuable for the study of cell-based therapeutic strategies in CKD and hypertension. However, the development of hypertension and renal injury induced by SNX in combination with a high salt diet has not been explored in immunodeficient athymic Foxn1 mice, a strain commonly used in xenogeneic transplantation. We studied the role of the immune system in relation to salt-sensitive hypertension in SNX mice, hypothesizing that T-cells and a high salt diet are required for the development of both hypertension and albuminuria.

## Methods

### Animals

Male wild-type (WT) immunocompetent CD-1 (own breeding colony) and athymic (AT) immunodeficient CD-1 Nude mice (Crl:CD1-Foxn1^nu^) from Charles River, 9–11 weeks of age were group housed in a light-, temperature- and humidity-controlled environment under standard conditions i.e. a 12 h light–dark cycle and with free access to water and standard chow. Protocols were approved by the Animal Ethics Committee of Utrecht University. Animal experiments were performed according to ARRIVE guidelines.

### Flow cytometry

T- and B-cells in the blood of WT and AT mice were counted using flow cytometry (Additional file 1) and confirmed the lack of T-cells and presence of B-cells in AT mice (Additional file 1: Figure S1).

### Experimental setup

Subtotal nephrectomy (SNX) was performed as previously described [9]. Via a retroperitoneal incision, the right kidney was excised and weighed. One week later approximately 2/3 of the remaining left kidney was removed. WT and AT mice either received standard diet or high salt diet (HSD, ground chow supplemented with 4–6% NaCl (CRM (E) FG; Special Diet Services Ltd., Witham, Essex, UK)), which was started one week after SNX (WT *n* = 7, WT + HSD *n* = 8, AT *n* = 16, AT + HSD *n* = 10). Four weeks after SNX, systolic blood pressure (SBP) was measured by tail-cuff sphygmomanometry; 16-h urine samples were collected by placing mice individually in metabolism cages, and blood was sampled from the cheek plexus.

### Biochemical analysis

Urine albumin was measured with a mouse albumin ELISA (Bethyl Laboratories Inc. Montgomery, TX). Sodium and potassium excretion were determined by flame photometry. Plasma urea was measured by DiaSys Urea CT FS (DiaSys Diagnostic Systems, Holzheim, Germany).

### Renal morphology

Three μm sections were sliced of formaldehyde-fixed, paraffin-embedded kidneys. A periodic acid-Schiff (PAS) staining was performed to visualize renal morphology, including infiltrating cells, tubular fibrosis, glomerular matrix expansion and glomerulosclerosis.

### Statistical analysis

Data are presented as mean ± standard error (SEM). Flow cytometry data of WT and AT mice were compared using a t-test. A two-way ANOVA, two-way RM ANOVA with Student–Newman–Keuls (SNK) as post-hoc test or linear regression were performed where appropriate using SigmaPlot 12.3 (Systat Software Inc., San Jose, CA) to compare groups and determine effects of the immune status and diet. A non-parametric Kruskal-Wallis test was performed on renal fibrosis data. *P* < 0.05 was considered significant.

## Results

### Baseline characteristics

At the start of the study, baseline measurement for body weight and SBP were performed on standard chow. Body weight of WT mice was significantly higher compared to AT mice (36.4 ± 0.7 vs. 26.7 ± 0.6 g, *p* < 0.001) while SBP of both groups was comparable (96.7 ± 3.9 vs. 96.2 ± 4.0 mmHg, *p* > 0.05).

### HSD increased SBP and albuminuria

**Fig. 1:**
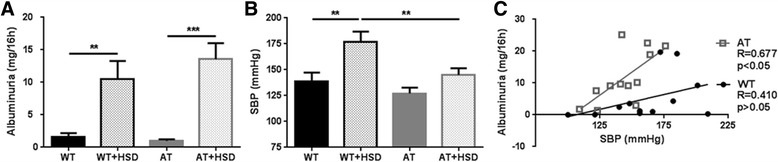
Albuminuria **a**, systolic blood pressure (SBP; **b**) and albuminuria vs. SBP **c** at four weeks after subtotal nephrectomy (SNX). WT: wild-type, AT: athymic, HSD: high salt diet. Mean ± SEM *p < 0.05, **p < 0.01, ***p < 0.001

HSD versus normal salt intake did not affect body weight of WT mice at week 2 and 4 after SNX (WT 37.6 ± 1.4 and 37.9 ± 1.7 g vs. WT + HSD 35.6 ± 0.9 and 37.0 ± 1.2 g, NS), but decreased body weight of AT mice (AT 29.6 ± 0.5 and 31.2 ± 0.5 g vs. AT + HSD 27.8 ± 0.5 and 27.4 ± 0.8 g, *p* < 0.05). Food intake for all groups was comparable at week 2 and 4 as assessed by potassium excretion (K excretion week 4, WT: 523 ± 66, WT + HSD: 618 ± 59, AT: 637 ± 38, AT + HSD: 560 ± 56 μmol/16 h, *p* > 0.05). The intake of salt was confirmed by a five-fold increase in sodium excretion in HSD groups (Na excretion week 4, WT: 354 ± 57, WT + HSD: 2951 ± 456, AT: 466 ± 40, AT + HSD: 2274 ± 270 μmol/16 h, *p* < 0.001). Independent of immune status HSD increased albumin excretion (*p* < 0.01; Fig. 1a). On a normal salt intake immune status did not affect SBP (WT: 139 ± 8 vs. AT: 127 ± 6 mmHg, *p* > 0.1). HSD increased SBP in WT mice (*p* < 0.01), but not in AT mice (AT: 127 ± 6 vs. AT + HSD: 145 ± 6 mmHg, *p* = 0.172; Fig. 1b). Consequently, SBP was higher in WT + HSD mice compared to AT + HSD mice (177 ± 10 vs. 145 ± 6 mmHg, *p* < 0.01). Immune status significantly influenced the relationship between SBP and albuminuria (*p* < 0.01), resulting in a positive correlation for SBP vs. albuminuria in AT mice (*R* = 0.677, *p* < 0.05), but not in WT mice (*R* = 0.410, *p* > 0.05; Fig. 1c). HSD did not induce significant differences in urea levels in either WT or AT mice (18.0 ± 2.2 vs. 24.3 ± 1.3 mmol/L and 22.1 ± 2.6 vs. 20.2 ± 0.9 mmol/L).

### Renal morphology

**Fig. 2:**
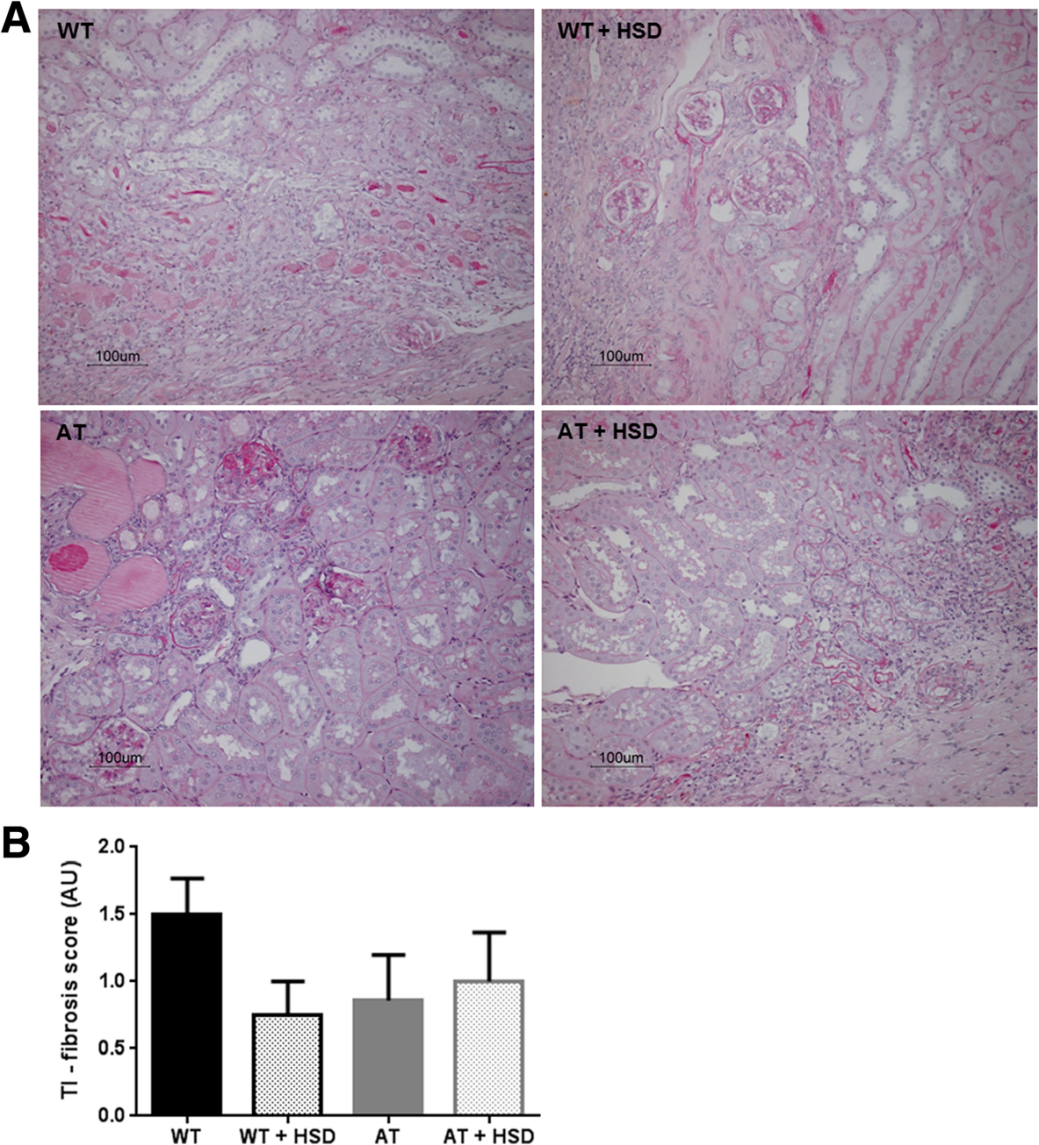
Representative photomicrographs of periodic acid-Schiff stained renal sections showing patches of severe inflammation and fibrosis in wild-type (WT) and athymic (AT) mice fed with standard diet or high salt diet (HSD) **a** and tubulo-interstitial fibrosis scores **b**

PAS-staining was performed to identify renal morphological damage. In all four groups patches of severe tubular inflammation and fibrosis were observed (Fig. 2a). Within those inflamed and fibrotic patches glomerular matrix expansion and glomerulosclerosis were also present. Severely damaged patches were alternated by normal to mildly injured renal tissue. Overall, the degree of tubulo-interstitial fibrosis was comparable between all groups (NS, Fig. 2b).

## Discussion

In the present study, WT SNX mice developed more pronounced hypertension in response to salt loading, compared to AT mice. However, both groups developed albuminuria to a more or less similar level. In this model tubulo-interstitial fibrosis was not related to the presence or absence of T-cells. Neither albuminuria and blood pressure were predictors for renal fibrosis.

The adaptive immune system, particularly T-lymphocytes, has been shown to play a major role in the development and maintenance of hypertension [3] and the response to salt load in various models of renal injury [4, 6, 8, 10]. Due to the pervasive effects of gene knock-outs in mouse models on various parts of the adaptive immune system, it is at present unclear which cell-type is the primary mediator of salt-mediated hypertension. The CD1-FoxN1^nu/nu^ mice employed in this study lack T-cells due a defect in the Foxn1 gene, which controls differentiation of the epithelial thymic meshwork [11]. The immunocompromised phenotype, therefore, arises due to a defect in the thymic niche, rather than an intrinsic defect in lymphocyte maturation. This is in contrast to Rag^−/−^ (Recombination-Activating Gene) immunodeficient mice, in which there is restricted B- and T-cell differentiation due to a defect in VDJ recombination [12].

The differences in hypertension between WT and AT mice observed in this study are likely primarily mediated by a deficient interaction between B- and T-cells. In a previous report, it has been shown that Rag^−/−^ mice, lacking both T- and B-cells, did not develop hypertension after AngII or DOCA (deoxycorticosterone acetate) salt. Transplantation of T-cells into these mice induced hypertension, whereas B-cells did not [13]. Conversely, in B-cell-activating factor receptor-deficient (BAFF-R^−/−^) mice, B-cells fail to mature, but the mice have normal numbers of T-cells. In these animals too, AngII induced hypertension was blunted compared to wild-type mice [14]. Transplantation of B-cells restored the development of hypertension. In the present study we show that FoxN1^nu/nu^ mice fail to develop salt- mediated hypertension, with a relatively isolated defect in T-cell maturation, but normal numbers of circulating B-cells. We, therefore, propose that T-cell dependent B-cell activation [15], may be important for the development of salt-mediated hypertension.

Our data suggest that AT mice are more sensitive to develop albuminuria on a HSD, as a lower SBP associated with a similar increase in albumin excretion as observed in WT mice on a HSD. This is consistent with previous observations that rats on a HSD with acute ischemia/reperfusion injury and treated with immunosuppressive drugs develop albuminuria, even though the immunosuppressive drugs completely blunt the development of salt-induced hypertension [8]. In contrast, in salt-sensitive Dahl rats treated with tacrolimus arterial pressure and albuminuria decrease in parallel [16], even though the number of T-cells was not even reduced by 50%. This suggests that in immunocompromised mice albumin leakage is not induced by systemic hypertension, but by increased permeability of the glomerular filtration membrane or an increase in glomerular hydrostatic pressure. Unfortunately, we were not able to study this in further detail due to lack of the required setup to perform sieving curves and micropuncture respectively. This could be noted as a limitation of our study.

## Conclusion

In conclusion, for the development of hypertension after SNX mature T-cells and a high salt diet are required. SNX induced albuminuria was independent of the presence of T-cells.

## Additional file

Additional file 1: Figure S1. Flow cytometry gating of T- and B-cells (A). T-cell (CD3, CD4 and CD8) and B-cell (CD19) markers in wild-type (WT) and athymic (AT) mice (B). (DOCX 379 kb)

## Abbreviations

AngII: Angiotensin-II
AT: Athymic
CKD: Chronic kidney disease
HSD: High salt diet
SBP: Systolic blood pressure
SNX: Subtotal nephrectomy
WT: Wild-type
