# Supplementary material for: T-cells contribute to hypertension but not to renal injury in mice with subtotal nephrectomy
Source: BMC Nephrol. 2017 May 8;18:153. doi: 10.1186/s12882-017-0555-0 (PMC5422945; doi:10.1186/s12882-017-0555-0)

**Title:** T-cells contribute to hypertension and renal injury in mice with subtotal nephrectomy

**Authors:** Nynke R Oosterhuis^1^, Diana A Papazova^1^, Hendrik Gremmels^1^, Jaap A Joles^1^, Marianne C Verhaar^1^

^1^Nephrology & Hypertension, University Medical Center Utrecht, Utrecht, Netherlands

**Supplemental data**

**Methods flow cytometry**

Whole mouse blood was blocked with FcR blocking reagent (Miltenyi, BergischGladbach, Germany) and stained with anti-mouse CD3-FITC (clone eBio500A2, eBioscience, Vienna, Austria), CD4-AF700 (clone GK1.5, eBioscience), CD8-APC (clone 53.6.7, eBioscience) and CD19 eFluor 450 (clone eBio1D3, eBioscience). After 30 min incubation in the dark, blood was lysed using an NH_4_Cl based lysis-buffer and cells were directly acquired on a BD FACS Canto II Flow Cytometer (Becton Dickinson, San Jose, CA). T-cells were identified by CD3, CD4 and CD8 positivity; CD19 was used as a B-cell marker (supplemental figure S1A). Cell counts were normalized to 1x10^5^ granulocytes, as identified on the FSC/SSC plot and confirmed the lack of T-cells and presence of B-cells in AT mice (supplemental figure S1B).

**Supplemental figure S1.** Flow cytometry gating of T- and B-cells (A). T-cell (CD3, CD4 and CD8) and B-cell (CD19) markers in wild-type (WT) and athymic (AT) mice (B).


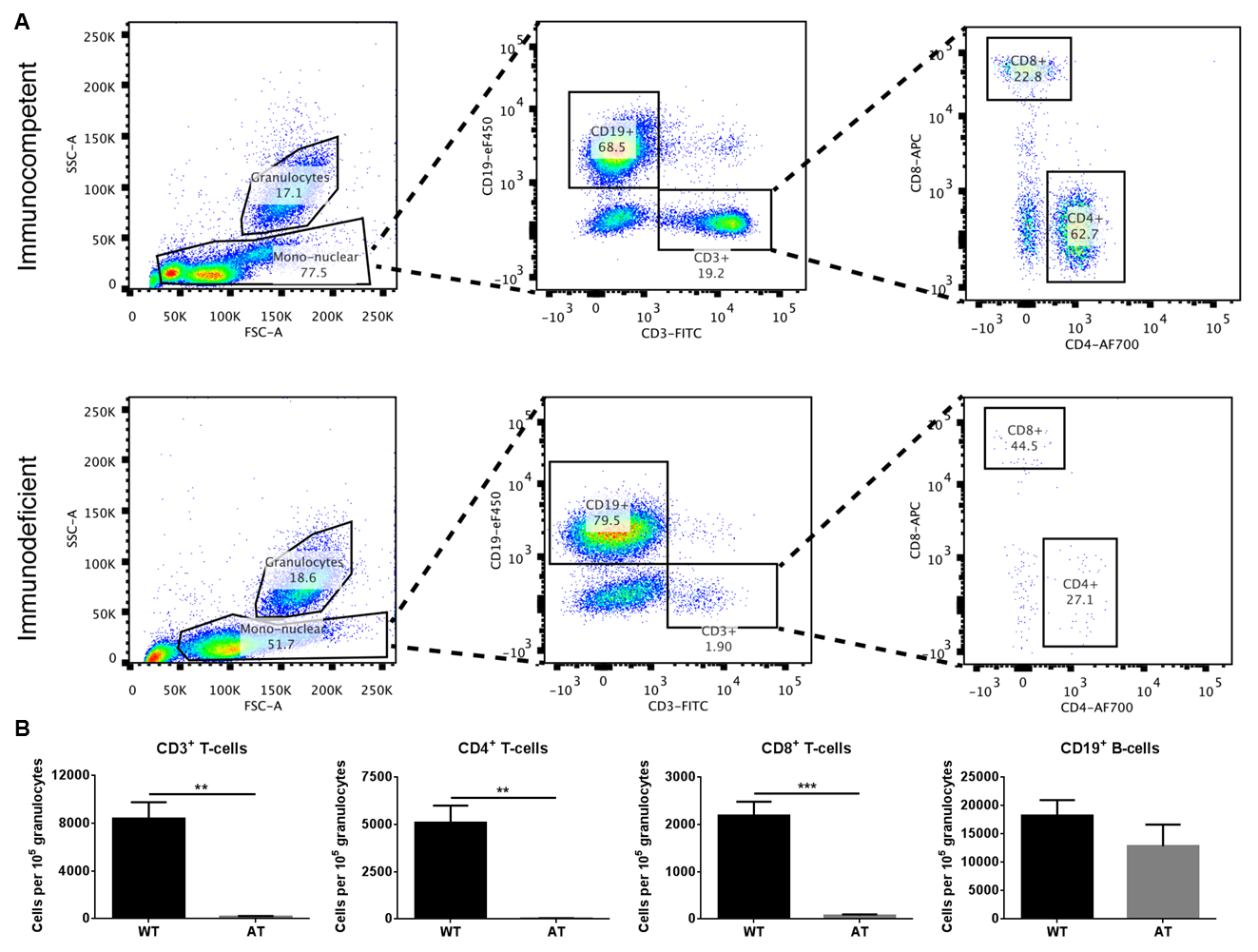

Supplement: Additional file 1: Figure S1. — Flow cytometry gating of T- and B-cells (A). T-cell (CD3, CD4 and CD8) and B-cell (CD19) markers in wild-type (WT) and athymic (AT) mice (B). (DOCX 379 kb) [file 12882_2017_555_MOESM1_ESM.docx]
